# Reporting of Foodborne Illness by U.S. Consumers and Healthcare Professionals

**DOI:** 10.3390/ijerph10083684

**Published:** 2013-08-19

**Authors:** Susan Arendt, Lakshman Rajagopal, Catherine Strohbehn, Nathan Stokes, Janell Meyer, Steven Mandernach

**Affiliations:** 1 Department of Apparel, Events, and Hospitality Management, Iowa State University, Ames, IA 50011, USA; E-Mails: lraj@iastate.edu (L.R.); cstrohbe@iastate.edu(C.S.); nmstokes@iastate.edu (N.S.); jrcmeyer@iastate.edu(J.M.); 2 Iowa Department of Inspections and Appeals, Des Moines, IA 50319, USA; E-Mail: steven.mandernach@dia.iowa.gov

**Keywords:** foodborne illness, diagnosis, healthcare professional, consumer

## Abstract

During 2009–2010, a total of 1,527 foodborne disease outbreaks were reported by the Centers for Disease Control and Prevention (CDC) (2013). However, in a 2011 CDC report, Scallan *et al.* estimated about 48 million people contract a foodborne illness annually in the United States. Public health officials are concerned with this under-reporting; thus, the purpose of this study was to identify why consumers and healthcare professionals don’t report foodborne illness. Focus groups were conducted with 35 consumers who reported a previous experience with foodborne illness and with 16 healthcare professionals. Also, interviews with other healthcare professionals with responsibility of diagnosing foodborne illness were conducted. Not knowing who to contact, being too ill, being unsure of the cause, and believing reporting would not be beneficial were all identified by consumers as reasons for not reporting foodborne illness. Healthcare professionals that participated in the focus groups indicated the amount of time between patients’ consumption of food and seeking treatment and lack of knowledge were barriers to diagnosing foodborne illness. Issues related to stool samples such as knowledge, access and cost were noted by both groups. Results suggest that barriers identified could be overcome with targeted education and improved access and information about the reporting process.

## 1. Introduction

### 1.1. Foodborne Illness Outbreak Data and Reporting Structure in the United States

A report by the Centers for Disease Control and Prevention (CDC) in 2011 [1] estimated that close to 48 million people annually in the United States contract a foodborne illness. During 2009–2010, a total of 1,527 foodborne disease outbreaks (675 in 2009 and 852 in 2010) were reported [2]. Numbers from the Foodborne Disease Active Surveillance Network (FoodNet) 2012 preliminary surveillance report [3] show only 19,531 cases of foodborne illnesses caused by any of seven bacteria and two parasites commonly transmitted through food were confirmed that year from a 10 state area populated by 15% of the U.S. population, or 46 million Americans. Due to the number of pathogens and the variety of reporting systems, it is difficult to determine the exact number of foodborne illness cases actually confirmed each year.

Surveillance data and reports of foodborne illnesses and outbreaks are submitted to the CDC and other government agencies from state and local health departments using several reporting systems. Some of the systems that have been used for decades track specific pathogens that are likely to be transmitted through food. The newer surveillance systems, such as FoodNet, have helped improve the quality, quantity, and timeliness of foodborne disease data but the CDC reports that these statistics reflect only a fraction of cases that actually occur. Under-diagnosis and under-reporting of foodborne illnesses present challenges for surveillance and the detection of outbreaks [4].

### 1.2. Consumer Food Safety Concerns

Brewer and Rojas [5] evaluated consumers’ attitudes regarding the safety of the food supply and noted general levels of concern with food safety increased with concerns about chemical, microbiological and regulatory issues. Only about 15% of the consumers surveyed in this study thought they had contracted a foodborne illness during the last year; more than 40% of these thought the source was restaurant food and less than 25% thought the source was a school or church event. Although these consumers were very concerned about the inspection of imported foods and restaurant sanitation, they did not assign a high priority to funding of regulatory issues such as hiring more inspectors. In addition, the majority of the consumers surveyed thought foods approved by the Food and Drug Administration were safe to eat (genetically modified foods (80%), irradiated foods (77%), food from animals treated with hormones (72%) or treated with antibiotics (74%)); however, about 30% reported they would not purchase these and more than 20% indicated they had reduced consumption. These findings show that to some consumers, production practices are a greater perceived food safety threat than safe food handling practices.

CDC data suggests there is under-reporting of foodborne illnesses by consumers [2]. This may be due to consumers’ lack of knowledge about safe food handling practices and resultant consequences when these practices are not followed, as well as misplaced concerns regarding source of safety risks. This was corroborated with a review of food safety issues conducted by Wilcock, Pun, Khanona, and Aung [6] that found much variation among consumers and their attitudes toward the safety of food, with a conclusion that there is a lack of knowledge about where and how foodborne illness can occur, and what to do in the event one becomes ill.

In a study of California consumers, most were confident in the safety of the food supply, with over 90% confident of the safety of fresh produce. Most were also confident of their food safety knowledge and that food handling behaviors were correct; yet findings suggested a need for directed consumer education [7]. This work built on findings of Williamson, Gravani and Lawless [8] who reported most consumers believed their foodborne illness had been caused by food prepared somewhere other than home. Knight, Worosz and Todd [9] interviewed over 1,000 adults in the United States to assess their perceptions of food safety at restaurants compared to other sectors of the food industry and found most think about food safety particularly when eating at restaurant establishments. A majority of these consumers stated that although restaurants were doing a good job, were capable, and were committed to food safety, this segment of retail food ranked lower when compared to other segments of the food chain (farmers, food processors and manufacturers, and grocery stores). The findings reinforced the importance of food safety behaviors at restaurants, particularly in areas of staff personal hygiene, workplace sanitation, and food handling.

In a 2011 National Public Radio (NPR) funded study of approximately 3,000 Americans to gauge attitudes and opinions about the safety of the food supply, the majority (57%) indicated concern about the safety of food, which was a decline from the 61% who reported concern in a 2010 study. However, an increased percent of respondents (11% from the 105 in 2010) said they had become sick from something they ate in the last three months. The food commodity which generated the greatest concern was meat by 44% of those polled. Fresh produce also was identified by 30% [10]. As noted in the Food and Drug Administration’s (FDA, 2000, 2004, 2009) [11,12,13] observations of risk factors for foodborne illness in hospitals, nursing homes, elementary schools, fast-food and full-service restaurants, and retail foodservice, there was high rate of noncompliance for three categories of risk factors: improper holding/time and temperature; poor personal hygiene; and contaminated equipment/prevention of contamination. Proper hand washing was the practice with the highest out-of-compliance rate for all facility types in all three of these FDA studies (2000, 2004, 2009) with higher compliance in noncommercial institutional settings than in commercial operations. This finding was replicated in an observational study of hand washing practices in four sectors of the foodservice industry [14].

### 1.3. Healthcare Professionals’ Roles and Knowledge

Healthcare professionals play an important role in the communication of public health messages to patients. A 2004 study by Hesse *et al.* [15] found that despite the availability of various online resources, around 62% of patients highly trusted physicians for all health-related information, including food safety. It is critical for healthcare professionals to communicate foodborne disease information to all patients especially those more susceptible to these types of illnesses (elderly, pregnant women, immunocompromised, and children). However, counseling patients on food safety is not among the top concerns for physicians which could be due to their lack of knowledge about food safety [16], lack of time, or perceived lack of benefit of providing such information to patients [17]. Physicians’ perceptions about the seriousness of foodborne illness, their perceived role as the information provider, and comfort with providing food safety recommendations plays a role in the likelihood of discussing food safety with their patients [16]. Semi-structured interviews conducted with healthcare providers working with pregnant women revealed that only 8 of 23 healthcare providers currently provided food safety information to their pregnant clients [18]. Healthcare providers reported confidence when providing foodborne illness prevention information, yet were not comfortable about their knowledge of foodborne illness or its treatment and diagnosis [19].

Among healthcare providers, registered nurses (RNs) and registered dietitians (RDs) may be better positioned to counsel patients about food safety than physicians; they can spend more time interacting with patients than physicians. However, Buffer, Medeiros, Kendall, Schroeder, and Sofos [20] found gaps in food safety knowledge and understanding among RNs and RDs about *Listeria monocytogenes*, a deadly foodborne pathogen, and one of particular concern for pregnant women. Registered dietitians were found to have more training than RNs in safe food handling and were more likely to provide comprehensive food safety messages to their highly susceptible clients; however, consistency in providing food safety information was lacking among both groups. In focus groups conducted with healthcare providers and caregivers for older adults, Wohlgenant, Cates, Godwin, and Speller-Henderson [21] found physicians were less interested in discussing food safety with older adults than RNs, nurse practitioners, physician assistants, and home healthcare providers. Medeiros, Chen, Hiller, and Kendall [22] found that cancer patients preferred receiving food safety information primarily from physicians or nurses, but were likely to follow the advice of their nutritionist, nurse, physician, or any other professional qualified in food safety. Therefore in-depth and frequent training of these personnel in foodborne illnesses prevention and treatment is critical for the communication of food safety messages to all types of audiences. Irrespective of the type of patients (children, elderly, pregnant or immunocompromised), healthcare providers can serve as an important source of food safety information thereby assisting in the prevention and timely diagnosis of foodborne illness.

## 2. Methods

### 2.1. Data Collection and Study Population

#### 2.1.1. Consumers

In the first phase of the research, three focus groups were conducted with consumers recruited from the general public from three cities in one Midwest state. Participants were recruited through a variety of methods such as newspaper advertisements, flyer postings in grocery stores, churches, colleges, and electronic newsletter postings. All participants signed an informed consent and completed a brief questionnaire that assessed participant attitudes about food safety and collected demographic information (e.g., age, gender, income level, employment status, ethnicity, and education level). Each participant filled out a name tag with a pseudonym of their choice to protect personal identity during the focus group and data analysis. All focus groups were conducted in English.

At the beginning of each focus group, introductions and ground rules were given to all participants by the moderator. Focus groups were moderated by an experienced moderator and supported by an assistant moderator. Each focus group lasted approximately 90 min. All the focus groups were audio recorded. Participants received US$40 as a “thank you” for their time. The representative research team members debriefed for approximately one hour following the focus groups.

For the consumer group, the words “food poisoning” was used as compared to foodborne illness because this term was found to be more commonly used in other education efforts, such as those at Mayo Clinic, WebMD, and CDC web sites [23,24,25]. Sample focus group questions are as follows: (1) How did you know you had gotten ill from the food and not something else? (2) What did you do? Did you report it and if so, to whom? (3) What could make it easier to report an illness? (4) How hard do you think it is to collect a usable stool sample?

#### 2.1.2. Healthcare Professionals

In this phase of the project, four focus groups were conducted with individuals recruited from healthcare facilities from four towns/cities in one Midwest state. Participants were recruited primarily through posting of flyers at appropriate work sites or newsletters with assistance from University Extension and Outreach staff. At the focus group session, each participant signed an informed consent and completed a brief questionnaire that assessed their attitudes about food safety and food poisoning, and collected demographic information (age, gender, income level, employment status, ethnicity, and education level). Each participant utilized a pseudonym of their choice to protect participant identity during the focus group and for data analysis. All focus groups were conducted in English. Because only two individuals committed to one of the focus groups, that focus group was handled as phone interviews while the other focus groups were all done “in person”.

At the beginning of each focus group, introductions and ground rules were given to all participants by the moderator. Though the participants had completed the informed consent form, participants were assured verbally that all information collected would be confidential and that participation was voluntary. Focus groups were moderated by an experienced moderator and supported by an assistant moderator. Each focus group lasted 60–90 min. All focus groups were audio recorded. Participants received US$40 as a “thank you” for their time. Questions asked in the healthcare provider focus group were similar to those in the consumer group. Three sample questions are as follows: (1) How concerned are your patients/clients about the safety of our food? (2) Where do you believe patients/clients have the greatest risk of acquiring a foodborne illness? (3) What are the barriers to diagnosing a foodborne illness?

To capture data from healthcare professionals not included in the focus groups (e.g., physician assistants), individual interviews were conducted. Those individuals who work with patients and have authority to diagnose foodborne illness were interviewed via phone. Participants for interviews were primarily recruited from focus group contacts. As part of the interview, participants were asked a series of close-ended questions concerning their attitudes toward food safety and foodborne illness as well as demographic questions. Open-ended questions concerning involvement and experience with diagnosing and treating foodborne illnesses were also asked. Interviews were audio recorded and transcribed verbatim.

### 2.2. Data Analysis

Descriptive statistics (frequencies) were compiled for data gathered from the focus group questionnaire. Recordings of all focus groups were transcribed verbatim. Transcripts were independently analyzed by at least three researchers to identify emerging themes. Individual analyses were then complied to form a consensus on the themes. Barriers to reporting foodborne illness and methods to improve reporting of foodborne illness were identified. These qualitative data analysis procedures are consistent with those discussed by Saldaňa [26]. For the individual interview data, two researchers independently reviewed the transcripts to identify emergent themes. The researchers then compiled the analyses and came to a consensus on the identified themes.

## 3. Results

### 3.1. Consumer Results

Table 1 presents the compiled demographic data from all three consumer focus groups. In summary, there were 35 participants who attended one of three focus groups (each focus group ranged between 11 and 13 participants). The majority was female (74.3%). Diversity in age and income level was achieved; 14.3% were between the ages of 18–25 years, 20% were between the ages of 26–34 years, 17.1% were between the ages of 35–49 years, 28.6% were between the ages of 50–64 years and 20% were between the ages of 65–80 years. The largest percentage of respondents had annual incomes less than $25,000 (37.1%) or between US$25,000 and US$49,900 (31.4%).

**Table 1:** Demographics: Consumer participant profile (*n =* 35).

Characteristic	Frequency	%
**Gender**		
Female	26	74.3
Male	9	25.7
**Age**		
18–25 years old	5	14.3
26–34 years old	7	20.0
35–49 years old	6	17.1
50–64 years old	10	28.6
65–80 years old	7	20.0
**Education**		
Some high school	1	2.9
High school diploma	5	14.3
**Some college**	11	31.4
Bachelor’s or Associate’s degree	11	31.4
Graduate degree	7	20.0
**Income**		
less than US$25,000	13	37.1
US$25,000–US$49,900	11	31.4
US$50,000–US$99,900	6	17.1
US$100,000–US$150,000	3	8.6
more than US$150,000	2	5.7
**Ethnicity**		
African-American or Black (Non-Hispanic origin)	2	5.7
Asian	2	5.7
Caucasian/White	30	85.7
Multiracial	1	2.9

Table 2 presents the compiled food safety questionnaire data from all three focus groups. The majority of participants had private insurance paid for by their employer (39%) or private insurance paid for by themselves (14.6%). About 17% of participants were on Medicare, 7.3% were on Medicaid, and only 4.8% didn’t have any insurance. Four of the participants (11.4%) indicated that they had a food allergy. Participants showed varied concern for the safety of food purchased to prepare at home with 11.4% not at all concerned, 28.6% not very concerned, 17.1% somewhat concerned, 14.3% concerned, and 25.7% very concerned. Participants concern for the safety of food prepared away from home was not as varied with the majority being somewhat to very concerned; somewhat concerned 28.6%, concerned 25.6%, and very concerned 31.4%.

**Table 2:** Consumer food safety questionnaire results (*n =* 35).

Question	Frequency	%
**Type of healthcare plan**		
Private paid by employer	16	39
Private, paid by myself	6	14.6
Medicaid	3	7.3
Medicare	7	17
None	2	4.8
Other	7	17
**Do you have a food allergy?**		
Yes	4	11.4
No	28	80
**Concern about safety of food purchased to prepare at home**		
Not at all concerned	4	11.4
Not very concerned	10	28.6
Somewhat concerned	6	17.1
Concerned	5	14.3
Very concerned	9	25.7
**Concern about safety of food prepared away from home**		
Not at all concerned	1	2.9
Not very concerned	3	8.6
Somewhat concerned	10	28.6
Concerned	9	25.7
Very concerned	11	31.4
**Type of food that poses the greatest risk for food poisoning**		
Meat	27	77.1
Seafood	29	82.9
Dairy Products	15	42.9
Fresh Produce	12	34.3
Packaged Foods	6	17.1
Eggs	18	51.4
Grains and rice	1	2.9
**Best approach to reduce the risk for food poisoning**		
More inspections	16	45.7
Better quality control	27	77.1
Stiffer penalties	7	20
Increased government oversight	6	17.1
Better consumer education	22	62.9
**In the past 3 months have you become ill from something you ate?**		
Yes	12	34.3
No	22	62.9
**For this illness did you seek medical treatment?**		
Yes	1	2.9
No	13	37.1
**For this illness, what type of treatment did you seek?**		
(Some participants provided multiple responses)		
Emergency Room	1	2.9
Doctor’s Office	3	8.6
Self-treated with over the counter medication	7	20
Other	1	2.9
**What did the medical provider do?**		
Took blood	1	2.9
Asked for a stool sample	1	2.9
Prescribed medication	1	2.9
Recommended increased fluid intake	2	5.7
Recommended rest	1	2.9
Nothing	1	2.9
Other	2	5.7
**Have you ever gotten sick from something you ate as an adult**		
Yes	32	91.4
No	3	8.6
**Did you seek medical treatment?**		
Yes	11	31.4
No	20	57.1
Both	1	2.9
**As an adult have you ever been asked to provide a stool sample**		
Yes	16	45.7
No	18	51.4
**Did medical provider confirm diagnosis with stool sample?**		
Yes	5	14.3
No	23	65.7
Both	1	2.9

Meat (27%), seafood (29%), and eggs (18%) were identified by participants as the types of food that pose the greatest risk for food poisoning. Participants also identified better quality control (27%), better consumer education (22%), and more inspections (16%) as the best approaches to reducing the risk of food poisoning. Twelve (34.3%) of the participants indicated that they had gotten ill from something they ate in the past three months and only one (2.9%) of those participants sought medical treatment for the illness. Thirty two (91.4%) of the participants indicated that they had gotten sick from something they ate as an adult and eleven (31.4%) sought medical treatment. One (2.9%) participant had blood taken, one (2.9%) was asked for a stool sample, one (2.9%) was prescribed medication, two (5.7%) were recommended to increase fluid intake, and one (2.9%) was recommended rest by their medical provider. As an adult, 16% of the participants had been asked to provide a stool sample and five (14.3%) of the participants were diagnosed with food poisoning through a stool sample.

In the consumer focus groups, participants were asked questions about how they knew it was food poisoning and what they did about it. Additionally, specific questions were asked about reporting food poisoning as well as seeking medical treatment. Methods or ways to get information about how consumers can report food poisoning were discussed. In addition, participants were asked questions regarding concern and difficulty with taking stool samples.

Major themes that emerged were related to symptoms presented (severity and duration); Table 3 provides the major emergent themes in each of the question areas. Sample illustrative quotes are provided to help in understanding the theme name provided.

**Table 3:** Identified themes and illustrative quotations from consumer focus groups.

Questions	Themes	Sample Illustrative Quotations
*How did you know you had gotten ill from the food and not something else?*		
	Symptoms: Severity and Duration	I think the symptoms, the, the diarrhea and the stomach pain and the, and the worst case is throwing up also. I was sick. I got really sick. Missed several days of work.
	Symptoms: Time after eating	We were eating at a restaurant, and within a few hours we were all violently sick. And about three the next morning, I just started retching and it went on and on for about three hours.
	Not like other illnesses	In the past when we’ve had the stomach flu, it’s not been like this. My daughter was puking every fifteen minutes for...a very, very long time so it just seemed very different than anything we’ve had before When you have the flu or something, you don’t, you feel kinda funny the whole week. But I was perfectly back to normal within twenty-four hours, or even less…
	Others got ill too	We all went out to dinner for a family dinner, and three of us had exactly the same symptoms at almost exactly the same time. And I was at a restaurant with friends. And there were four of us. We shared the appetizer, and then we all got sick the next day.
	Diagnosed as FBI	And I was diagnosed with cryptosporidium which *can* be a foodborne illness. Dramatic. Stool sample and diagnosed and confirmed. It was E. coli. It took them three months to get it finally out of my system.
	Only food eaten/no other diet changes	But I have a pretty routine diet. And when I ate something m-, that...I went out to eat...I had the runs for an extended period of time. It wasn’t concerned... that’s the only thing that I could appoint it to. I hadn’t ate all day. And then, we got tacos from a taco place in town, and I mean, I saw that the meat was pink but I figured it would be OK, and I went ahead and ate it. The first and last time I ate pink meat.
*What did you do?*		
	Dealt with illness/suffered through/self-medicated	I just waited it out...at home… I was so sick…you know if you’re dry heaving and everything, you just feel like the lining of your stomach is coming out of you. But I was too busy throwing up and dying that I just... ...I just said, “No, that’s fine. I’ll just suffer.”
	Contacted a healthcare professional	And on the second or third day I went to the doctor, and I did take a stool sample, and it was diagnosed as salmonella. And I went up there (hospital). They took the stool sample, and took some blood work, and he said that I had food poisoning.
	Contacted food provider	Yeah, I reported it. I reported it to the restaurant. Other than going to the convenience store with that empty package and doing a little bitching... that was my only reporting. I laid in bed for two days.
*Did you report it and if so, to whom? Why wouldn’t you report a potential illness?*		
	No: didn’t know who to report it to or did not think to contact someone	The first time that it happened to me, I never considered it, you know. Who would I report that to? Where would that go? I wasn’t gonna go back to that restaurant ever again… I didn’t want anything from them, so I didn’t bother with that. But it never even crossed my mind to contact someone else. I think it’s not knowing...I mean, I guess that my...the other reason was, I didn’t know if I should contact the restaurant themselves. I didn’t think that that would have done anything. But it also didn’t cross my mind to contact the health department. I don’t know why.
	No: too ill	And it burns and it’s just...bad. You have to like sit at, sit in the bathroom and have a garbage can in front of you. No, because my wife and I...the only thing we had the same that night was soup. And we both, within a half hour, were down. We were down for two days. And there was no leaving the house or anything just to go to the doctor.
	No: not sure where it (food) came from	I was like ninety-seven percent sure that I got it from that restaurant... but...if I’d been one hundred percent sure, then I would’ve called without a doubt but...I was sort of talked out of it by other people who thought maybe it could have been something else. Yeah, ‘cause you’re making a serious allegation if you’re saying that someone caused you to be sick. If there’s... if you don’t have proof, you could be wrong.
	No: not sure it was food poisoning	I think from like a health standpoint, most people don’t report like getting sick because the symptoms, it could be anything else like from a cold to a flu and so most people always assume the least of the issues and don’t really do anything about it… Or it might not necessarily be what got you sick…if you don’t a hundred percent know. ...if I’d been one hundred percent sure, then I would’ve called without a doubt but...
	No: wouldn’t do any good to report it or is not worth it to report it	I guess the only other thing that I would have to say is that, you know, when we take time to do something we’re always weighing out, is the time that we put in worth whatever outcome comes out? And so, we were traveling. We weren’t sure. So it’s like the time and energy and everything that it would’ve taken to report it at that time, we didn’t feel, was worth like whatever potential outcome we had. And then you’re thinking, once it’s over, well, I really don’t wanna do it now because... you know, what benefit is it to you personally? I did have the nagging thought, “Maybe I should go ahead and do it just to confirm it. Just so that there could be a confirmed report.” But, again, there wasn’t that personal—it sounds really selfish—but there wasn’t that personal incentive because it was done.
	No, not convenient	And I didn’t report it because, I was traveling back here and we were on the road and stuff. No insurance
	Yes: reported it	I actually had a friend tell me, “Why don’t you report it?” And I go, “Oh! I, I didn’t think of that!” So first I called First Nurse because I was concerned about my daughter. She was the one who was the most sick, and, and then she said the same thing, “You need to report it.” And I don’t remember the number she gave me ‘cause I was really sick at the time, but whatever number she gave me, I called in and reported it. I called the FDA on ‘em. I’ve got sick there on three separate occasions… all three times it was for two days...at a time.
*What would make you seek medical treatment if you suspected you had gotten sick from eating a food?*		
	Length/duration of illness	If it was over a week at this point, maybe I would think, OK, if it was over a week, then I should go to the hospital. …for me, that’s more than twenty-four hours. If it wasn’t subsided, then I’d definitely do something about it
	Severity of illness	And they threw up every fifteen minutes like all night long. And they were pretty sick. But First Nurse really encouraged us to...to look for the signs of dehydration and just some of the warning signs that things weren’t going well. If I had like blood like in vomit or a stool or anything like that or really dehydrated, then I definitely would go seek medical attention.
	Child	I think if a smaller child is involved, I think that I would seek help... Yeah, we called First Nurse because my daughter and my son were both sick, and they’re ten and six.
*Who would you report it to?*		
	Poison Control	The more informed, I think the general public has a lack of knowledge what to do in those cases. Like most people wouldn’t know to call the poison control, you know. Like Poison Control. I didn’t even think about that. Is that something... ‘cause I think, when I think Poison Control, I think if you swallow something you’re not supposed to swallow.
	FDA	I would think the FDA. You see signs on, on the outsides of buildings that give them grades on, on their food quality. Either the FDA or...Poison, Poison Control possibly
	CDC	The doctor reported my case, and the CDC representative contacted me.
	Healthcare provider	If you don’t know who to report it to, you could always do...what I did, just call your doctor and...talk to a nurse, and they’ll tell you...who you should report it to.…for me it’d be my healthcare provider...just because if it’s bad enough that I’m going there... if they had brochures or if I could seek advice or something like that.
	Restaurant/Grocery Store	I think it’s the responsibility of the restaurant or the grocery store. I would definitely report it to them, and it’s their responsibility to get the word out to the proper authorities and customers to, ‘cause they’re the ones like right there at the center. They can stop selling or producing the thing right then and there. I would definitely at least talk to a manager-, like a higher up, like the manager of the restaurant if I got sick there.
*What would be the best way to get them (focus group participants) information about how to report food poisoning? What would make it easier to report?*		
	Telephone number	I think just...an 800 number or, I mean, I would probably… I would just contact the Department of Public Health and... I don’t know...an easy 800 number or an easy person that I could talk to at the Department of Public Health. I think it has to do with exposure…what we were just talking about...because as far as exposure, you hear about 1-800-BETSOFF. But you ain’t never heard nothing about food 1-800-THROWUP
	Internet	I’m more likely to want to fill out a form online...rather than call. ...the younger generation spends a lotta of their time on computers. So, of course, a lotta time is spent on Facebook …having something online that would be easily accessible to type in...“I got sick” and…
	Media (TV, Radio)/National Campaign	I would think a public service campaign or something that would tell people that it’s not, if you get ill and you think it’s from food, it’s not your responsibility to go to the restaurant and take care of it. It’s to call Public Health. Yeah, with an 800 number. But I do think that it would be a good idea for, for you know, somebody to make a PSA (public service announcement), have it on the TV stations, and say, “Hey, you know, if, suspect you have food poisoning,” ...my opinion is, the media doing a PSA.
	Restaurant	Yeah, if they could even put up posters up at restaurants. … maybe the easiest way would be to put it on those FDA signs. They’re on every building that serves food.
	others	Put it on the packaging if it’s...prepackaged food. And if you have suspected food poisoning or if you’re diagnosed with food poisoning, you give ‘em the brochurewhen they leave the hospital or the doctor’s office. And then it’s up to you as...the patient, to decide if you wanna call the health department and report it …
*What could make it easier to report an illness?*		
	How: education	Well, it comes to education also…. I mean, do people know what they should be reporting? Like what is a real sign of a foodborne illness? …..most of us aren’t gonna be able to say, “For sure, this is what happens”……getting the information out there to what should be reported. I think one thing that would’ve maybe helped people is...I didn’t realize that if I went to the doctor that they could actually tell me for sure if that’s what I had or not. And I think lots of people would feel more comfortable reporting it if they knew for sure…
	Knowing someone cared/listened/do something/held accountable/ something in it for me	So it’s like the time and energy and everything that it would’ve taken to report it at that time, we didn’t feel, was worth like whatever potential outcome we had.Cause I think we, in our mind, thought, Well, we’ll probably call the restaurant and they’ll say, Oh, we’re so sorry that happened or something and then what would the outcome be? I would say just thinking that somebody cares, and that someone’s gonna listen and that there might be some action taken care of.
	Knowing for sure it was an FBI	And then going to the doctor gives them that confidence to report it ‘cause I know for sure that’s what I had. And what...like what tell-tale signs you should be looking for. To make sure that if, it’s not just the flu or...but that it did come from a foodborne illness.
*How concerned would you be if the medical provider asked you to supply a stool sample? Why?*		
	Concerned: something bad/uncertainty	I think for me it’d be more of a concern of how serious is this because all the doctor’s visits I’ve ever gone to, they’ve never asked for that. So now that this is something new, it’s like does this mean it’s worse than everything I’ve encountered before? It would be scary at first to have your doctor probably say that, because my mind wouldn’t necessarily go to the food poisoning idea. It might go to a lot of other...areas.
	Concerned: inconvenient	How are you gonna set aside a whole another entire day just to point the finger at someone? my concern was I’m just too sick to do that.…But, when you’re that sick, you’re thinking, “I don’t wanna do it now because I don’t wanna leave the bathroom.”
	No Concern: previous experience	I wouldn’t be concerned. I wouldn’t want to, especially ‘cause my friends had to do it so I know how not...fun it is. Or if you’ve done stool samples before, I mean, some of us...it’s kind of routine with my doctor so I’m...I’m surprised you haven’t all been doing it…
	No Concern: this is way to diagnose/help you get better	I think if knowing that I did that it would help me feel better, I would probably do it. I was gonna say ‘cause I remember feeling like, “Oh, this is good. We’ll really get to the bottom of this.” And also that having missed a week of work, which put a big burden on to co-workers, it was kinda nice to say, “I have, the State of Iowa says I had salmonella.”
	What’s in it for me/benefit to me (cost/benefit)	...you know, what benefit is it to you personally? I did have the nagging thought (that) maybe I should go ahead and do it just to confirm it…But, again, there wasn’t that personal… incentive… I mean, if you’re sick for four or five days in a row, the moment you’re not sick you have a million things that you have to catch up with. How are you gonna set aside another entire day just to point the finger at someone?
	Concerned: embarrassed	I don’t think it’s a question of concern. It’s probably a question of maybe... embarrassment or something? Yeah, there’s gotta be a certain amount of embarrassment to any kinda private question like that.
*How hard/difficult do you think it is to collect a usable stool sample? Why do you think this?*		
	Difficult: previous experience	After having given one stool sample, I would almost just rather tell them to assume that whatever you think I have, I have it. Let’s deal with it! And skip the stool sample. You are at the most wretched of your being, and you are asked to then do this. I looked at it and said, “I don’t know if it’s worth it”. It was very complicated.
	Difficult: handling	Well, and I would think contamination! I mean, you think about it. I thought it was difficult because I was concerned with what was safe handling of the material.
	Difficult: messy/inconvenient/process and equipment	I think it might be slightly more difficult when I had food poisoning, though. I’ve never done it, but I would probably think the same thing. My friend said she had to use a trash can, so... she said it wasn’t very easy to do.
	Difficult: doctor’s office vs. home(and mailing)	I definitely think the worst part is having to go to the post office. And everybody knows what you’re doing with your box that’s going in to the University of Iowa. And I think that should be done at a medical facility. They give it to you to take home and do at home. I feel like that could very well, easily be handled in that facility right there when they’re asking you to provide that.
	Not Difficult: previous experience	And I’ve done it, and it’s not that difficult. I don’t think it would be too hard. They have these little hats that fit in the toilet so you wouldn’t have to worry about...fishing it out.
	Embarrassed	When it’s your own, it’s, it’s...the embarrassment factor is is up there. When you’re cleaning up after someone else, it’s their embarrassment, not yours. And, and our parents severely ingrained into our, our memories, at a very young age, that, Ooooh, that’s gross. It goes in the toilet and away from everyone else.

### 3.2. Healthcare Professional Results

Table 4 presents compiled demographic data from all healthcare practitioner focus group participants. All participants were female and Caucasian (except one missing response); 31.3% were between the ages of 26–34 years, 37.5% were between the ages 35–49 years and 31.3% were between the ages 50–67 years. The largest percentage of participants chose an “other category” to indicate their profession. The “other” category included office nurse and medical assistant, for example. The majority worked at medical clinics (68.8%) and was classified as full time employees (81.3%). 

**Table 4:** Demographics: Healthcare professional participant profile (*n =* 16).

Characteristic	Frequency	%
**Gender**		
Female	16	100.0
**Age**		
26–34 years old	5	31.3
35–49 years old	6	37.5
50–64 years old	5	31.3
**Profession**		
Nurse practitioner	1	6.3
Allied healthcare professional	2	12.5
Other	13	81.3
**Location of employment**		
Hospital	5	31.3
Medical clinic	11	68.8
**Employment status**		
Full-time	13	81.3
Part-time	3	18.8
**Ethnicity**		
Caucasian/White	15	100.0

Table 5 presents the compiled food safety questionnaire data from all healthcare practitioner focus group participants. Participants showed varied levels of concern about food safety with 6.3% not very concerned, 25% somewhat concerned, 50% concerned, and 18.8% very concerned. However, they perceived that their patients/clients were less concerned than they were. A majority indicated foods away from home (68.8%) as the greatest risk source for acquiring a foodborne illness. Participants identified better quality controls (68.8%), better consumer education (62.5%), and more inspections (56.3%) as the best approaches for the industry to use to reduce risk of food-borne illness. When asked how often they saw patients/clients with complaints due to something they ate, 50% of participants either never saw these patients or saw less than one per month. There were no participants seeing patients more than 10 times per month with complaints from something they ate. Five (33.3%) of participants reported they had had a patient/client who had a confirmed foodborne illness. Time between food ingested and treatment sought (93.8%), lack of patients/clients knowledge (87.6%), cost (50%), and unavailability of suspected food (50%) were identified as perceived barriers to diagnosing a foodborne illness. The participants also indicated e-mails (75%), newsletters (31.3%), webinars, and face to face communication (25%, respectively) as the preferred communication methods to receive updated information on diagnosis, treatment and reporting of food-borne illnesses. 

**Table 5:** Healthcare professional food safety questionnaire results (*n* = 16).

Questions	Frequency	%
**Your concern about the safety of our food**		
Not at all concerned	0	0.0
Not very concerned	1	6.3
Somewhat concerned	4	25
Concerned	8	50
Very concerned	3	18.8
**Your patients/clients’ concern about the safety of our food**		
Not at all concerned	1	6.7
Not very concerned	6	40.0
Somewhat concerned	4	26.7
Concerned	3	20.0
Very concerned	1	6.7
**Greatest risk for acquiring a food-borne illness**		
Home	5	31.3
Away from home (restaurant or other foodservice establishment)	11	68.8
**Best approach for the industry to use to reduce the risk of food-borne illness**		
More inspection	9	56.3
Better quality controls	11	68.8
Stiffer penalties	6	37.5
Increased government oversight	2	12.5
Better consumer education	10	62.5
Better healthcare provider education	8	50.0
**How often do you see patients/clients with complaints due to something they ate?**		
Never	2	12.5
Less than one time per month	6	37.5
1–2 times per month	2	12.5
3–10 times per month	6	37.5
**Have you ever had a patient/client who had a confirmed foodborne illness?**		
Yes	5	33.3
No	10	66.7
**Barriers to diagnosing a foodborne illness**		
Cost	8	50.0
Required tests	6	37.5
Lack of patient/client knowledge	14	87.5
Lack of healthcare provider knowledge	7	43.8
Unavailability of suspected food	8	50.0
Time between food ingested and treatment sought	15	93.8
Other	1	6.3
**Preferred communication method to receive updated information**		
E-mails	12	75.0
Webinar	4	25.0
Newsletters	5	31.3
Face to face, in person	4	25.0

In healthcare provider focus groups, participants were asked questions about their experiences with patients who had foodborne illnesses and it was noted that past experience (self or patient) with foodborne illness directed awareness. The healthcare professionals reported the following as reasons to suspect a foodborne illness: multiple people getting ill with similar symptoms, severity of symptoms, eating at a restaurant, length of time between eating and symptoms (varied from short to long). Treatment primarily addressed symptoms but diagnosis may not be done. When asked what tests or procedures are usually done when a foodborne illness is suspected, respondents reported stool sample, blood, and CAT scan. Some mentioned that the goal was to “rule out everything else”. Some participants appeared unaware that a stool sample was the method of diagnosing foodborne illness. It appeared the type of organization (e.g., hospital, nursing home, or clinic) drove how, when, and why stool samples are taken. For example, in hospitals, stool samples may be taken more often as the lab facilities are “in house”. Several barriers in getting stool samples were noted: no follow-through by patient, cost (dependent on patient population), patient lacking capability, not ordered because no foodborne illness suspected, and symptoms subside so no sample was taken. Table 6 provides the major emergent themes in each of the question areas. Sample illustrative quotes are provided to help in understanding the theme name provided.

**Table 6:** Identified themes and illustrative quotations from healthcare professional focus groups.

Questions	Themes	Sample Illustrative Quotes
*Tell me about your experiences working with patients with a suspected foodborne illness?*		
	Patient gets admitted	Not unless the severe case is where someone actually got admitted and that’s, you know, that’s pretty far and few in between that they’re actually admitted. So, it probably goes very, very unreported at least...where I’ve been aware. Or they wait ‘til they’re so bad dehydrated, they’re in the hospital.
	Asked questions to patient	When I have several come in, you know, I always start with, oh, you know, “What did you eat? Did you all eat the same thing? You know, did the person that ate this get sick? Or this one not get sick?” …kinda work down the list of who ate what… But...and you have to think about time of onset.
	Treat symptoms	Giving them IV fluids and trying to control… the vomiting and not wanting to stop, wanting em to get it …completely out of their system, but trying to make them comfortable at the same time. Yeah, generally our physicians will tell ‘em to do bed rest and liquids and wait it out three or four days and then come back for further testing.
	Nothing done	But with the fast pace, you don’t have that time to evaluate... I think sometimes with adults it gets passed off
*How did you know to suspect FBI?*		
	Patient: severity of symptoms	Well, the cases where I’ve seen it… more violent vomiting and more violent diarrhea and abdominal pain. And often they do… you pass out or the fainting goes along with it People that have gotten the E. coli, they’ve been so ill that they’ve even died or come very close to dying. And their symptoms are even more so, you know, the, the extreme bloody diarrhea…
	Onset of symptoms	Uncontrollable vomiting and diarrhea and said “you know what” I just ate at this place twenty min ago. Usually within the first couple hours that usually you eat.
	Symptoms: multiple people ill	When I have several come in, I always start with, oh, “What did you eat? Did you all eat the same thing? Did the person that ate this get sick?” If our triage nurses triaged several people with the same symptoms, then when they come in you would have that knowledge so you could ask them at that point if…they’ve all ate at the same place.
	Personal experiences with FBI	And I became, oh, violently ill, within about a ten, twelve hour region. Ended up passing out. I ended up being hospitalized because I got so violently ill. I will say that I personally had food poisoning one time myself for a, a chicken pizza at a restaurant.
	Patients: self-diagnose	And patients anymore are getting very savvy with the Internet and are trying to self-diagnose. “I already know what it is. I don’t need to come see you. I just need medicine.” When the physician doesn’t know what it is yet until we do testing! Hey, this is what happened. I wondered if it’s food poisoning?” And...I don’t know if it’s addressed right away as that.
	Not diagnosed right away, often have to come back to get tested or not tested at all	Generally our physicians will tell ‘em to do bed rest and liquids and wait it out three or four days and then come back for further testing. Patient came in and I know he was seen a second time then ‘cause he wasn’t getting any better. And then they did some testing...stool samples...and they found it out. It was salmonella.
	Reporting	I’ve never had a patient report it
*What did you do?*		
	Made Calls	In the Poison Center where they would think...that would probably, was more of a link than actually just the straight emergency room was the Poison Center would get the call….And it was more accessible than probably Public Health
	Ask questions to patient	And, you know, lots a times, whenever they go in and ask ‘em prompts ‘em to remember something so. I know the provider I work with, usually that’s the first word outta their mouth: “Well, who else at home is sick?” Or “Is, were you around anybody else that was sick?” Well, is anybody else at home sick? And they go, “Oh, no.” But then by the time the doctor gets in the room... they start to think about it. And then they go, “Oh, yeah, well so-and-so and so-and-so are whatever” or they don’t, or what they tell them is a totally different story than what they tell you anyway.
*What tests or procedures are usually done when you suspect a foodborne illness?*		
	Blood and stool	I would imagine it would be the basics. It’d be stool studies and possibly a CBC to see if it’s...either eliminate viral illness or just say it’s a viral illness Taken stool samples just for… to make sure it wasn’t something else or if they can test that.
	Other diagnostic tests	Straight sigmoid scopes...to check the intestine to make sure that the actual walls of the intestine are staying hydrated I do know, in a few cases, of actual rectal exams, just to make sure in case of parasite infestation *versus* foodborne illness.
*When are stool samples taken?*		
	Symptoms that trigger taking stool sample	When they’ve had the nausea and they’re having diarrhea frequent with stools. I think a lotta times it depends on how long they’re having the diarrhea for and how many episodes of the diarrhea they have a day.
	Vulnerable populations	The only time I think I’ve ever heard of that is in nursing home population, just because of the close proximity.Numerous episodes and lots of the kids were having blood in it. But we deal with babies on up and like I said, some of the babies are having blood in their stool. And sometimes that can indicate they’re not tolerating breast milk which could possibly be sorta foodborne in a way for a baby
	Difficulty with stool samples	…you do your own stool sample. And then you either bring ‘em back or mail ‘em back. We prefer patients to bring ‘em back… Maybe the literacy of the patient to understand how to do it. If they didn’t say that they would really be able to do it right at all and it wouldn’t be beneficial, so they find other ways to diagnose them.
*If a stool sample is not taken but a foodborne illness is a potential diagnosis, what are the likely reasons for not taking the stool sample?*		
	Cost	I mean, a lotta that stuff, even though it might be only fifteen or twenty dollars, a lotta the patients that we service, they don’t have that. And just like with this gentleman that came in, the reason he came in and the whole family didn’t come in, the ones that had that sickness, was because they didn’t have insurance.
	Lack of knowledge: Healthcare Professionals	I think maybe a little bit of both. Or, if they come in and they just say, “Ohhh, I’m not feeling well,” and maybe they don’t ...fully describe it or if they’re the only one that there is sick and... I’ve triaged lots of phone calls. And off the top of my head, I’ve never thought of foodborne illness. With food poisoning, is it ever to where they don’t have the diarrhea but they’re just vomiting? Would that be where they wouldn’t do stool samples? They might...I don’t know. Do they ever take like samples of the vomit? Or no? Or would it not be in that?
	Lack of knowledge: Patients	So there’s also that factor that you just don’t know that ten other people that walked outta that restaurant that night became ill. So you just say, “Oh, OK. Well, I musta picked up a bug somewhere.” So I think that’s the, and even our, ourselves, I think if I were to become sick, I’d think, “Well, I just have a bug.” We just don’t assume that, you know. I think, this goes back to patient education, patients who think they have foodborne illness, that means diarrhea. So if their stools become formed, at all, even if they’re still loose, even if they’re still very frequent, if they become formed at all, they feel, “Oh, it must not have been that.” Unless it stays the diarrhea, they don’t feel it needs to be tested.
	Patient: mess/embarrassed	And I think some people are just shy and embarrassed and don’t wanna bring back their stools. They don’t wanna touch that to smear it on a card.
	Stool sample collection challenges	Maybe the literacy of the patient to understand how to do it. If they didn’t say that they would really be able to do it right at all and it wouldn’t be beneficial, so they find other ways to diagnose them. The specimen containers themself, when you’re instructing a patient on proper collection procedures, the lids of the containers have spoons on them so that if the bowels are more formed, that they can actually use it to fill the container.
	Patient: non-cooperation	You can order everything you wanna order and their insurance’s gonna possibly pay for it. But if your patient’s mother or father or whomever doesn’t take them up to the lab to go get that stuff and get that done, you will never know what they had. I don’t know how many times a year a doctor would write that: “Come back with the stool samples “or” Once you can get a stool sample,” and I can guarantee you most of them didn’t come.
	**Treat Patient and move on**	But as you can see as a practitioner, you know, it’s just better to treat the symptoms and say follow-up if you don’t improve in the next couple days. Yeah. And they may walk in the door acting like they’re just so, you know, worried, worried, worried. But yet, once they got a little piece of paper with an antibiotic written on it or whatever, there was their fix. They were, they were satisfied and...bye, bye.
	Symptoms not severe	Possibly the beginning of the illness and then they waited too long to be treated. And it’s possible that the amount of active infection has already been fought, and they’re already on the downhill of it. When there’s just been nausea and vomiting and no diarrhea.
*What information or tools would aid you in doing your job related to patients with suspected foodborne illnesses?*		
	Patient information: flyers	Flyers about that to, that could kind of simplify how to take care of, you know, their foods in their own home and what, you know, to wash your hands. And it would be beneficial for our clinic is if we had it in Spanish too. Flyer, “if you suspect a FBI, please call this number”.
	Public education	You look at employees in the workforce…. Those that are hired in restaurants… do they really have the education. I think with the public, a lot of teaching food storage and how long to keep food for and all that. I mean, some people think you can keep it a week, some three days, you know. Like she said, some ‘til there’s mold on it.
	Healthcare professional knowledge	In talking about foodborne illnesses, we’re all relating to four or five different symptoms. And … there may be other things we should be looking for that we’re not identifying. In most foodborne illnesses, the time of ingestion to time of illness, what should we be looking for?
	Algorithm/decision making tool	Where you initiate getting the information about whether or not it is a foodborne illness at that decision-making point. So I liked the fact that it said, you know, ask ‘em if they have this, this, and this. If they say yes to these, you go down to here and you ask this lump of questions. It helps you narrow it down.
	Easy stool sample collection	But, you know, like a food, like just some kind of testing kit that if, you know, if you’re suspecting that, it has the equipment that you need right there as far as the lab slips and the, the tubes and the culture vials. So it is very difficult. And I can understand that, that’s why you would just give up, especially if you are feeling better. I don’t know how you could fix that because there’s only certain ways you can collect certain things.
	Web-based resources	If they (pamphlets) could be available for download for use, I think that would be a great help. Policies and procedures website where you could go to print off stuff.
*What do you think is the best way to get information to your fellow healthcare providers?*		
	CEU’s (continuing education unit)	Sounds like a good CEU topic. You know, what are the different...foodborne illnesses that we’re gonna see more commonly in this area? And what are the onset times and, and the symptoms they might display? And then, what do we do about it? You know, it just kinda leads you in, you know, into that process. Yeah. I don’t know if, it sounds to me like this would be a really good topic for some CEUs.
	Email	But I love getting the notices like through the email... If they wanted to do a short...day-by-day learning thing or, I know they could do some emailing.
	Face to face information sessions	Everybody learns differently. I mean, some people are, you know, visual learners. Some people are auditory. Some people read and they know it. getting information out, I think a lotta people do like to do these small groups. I know we do… I think it helps just to be…I don’t know, be able to, openly discuss things.
	Web-based resources	You know, that we could do this webinar or, you know, if your clinic is interested in setting up a time with... …go to this site, you know. This is where we’re gonna give all our patient information from this site and this site only.

In addition to the healthcare providers who participated in the focus groups, other healthcare providers (e.g., physician, physician assistant and registered nurse in charge of county public health office) were interviewed. Themes that emerged from interviews with these healthcare providers responsible for diagnosing foodborne illnesses (*n =* 4) included the following:

- Clear understanding of foodborne illness not indicated—how it might occur and what it might entail
- Clear understanding of food system controls and prevention not indicated
- Severity of symptoms appear to guide treatment and testing (further steps)
- Past experiences in responding to an outbreak and experiences of preceptors/mentors/trainers with an outbreak appear linked to raised awareness—not from formal education
- Food safety at the food service establishment level seems to be a concern or seen as the cause of foodborne illness rather than home
- Desired resources include more contact with Public Health Department (PHD) and resource to aid in easily identifying potential symptoms and causes

## 4. Discussion and Conclusions

It should be noted that the study was limited to 55 focus group members and interviewees in one state, therefore results should not be extrapolated to a much larger population without further study. Qualitative and quantitative data gleaned from focus groups and interviews with consumer and healthcare providers provides detailed information as to why foodborne illness may not be reported. Similar themes emerged from both stakeholder groups: a lack of awareness about foodborne illness risks and knowledge about the reporting process. Efforts to improve outreach information to consumers and healthcare providers about the importance of reporting along with specific instructions on how to do so should be reviewed. In the state in which data was collected, multiple agencies are involved with issues related to food borne illness. Thus the challenge for consumers and healthcare providers suspecting a “food related incident” is to navigate among various government organizations to identify action steps. In addition, consumers’ reluctance to report appeared to be due to an unwillingness to cause damage to the reputation of a foodservice. This finding may be a limitation of this study which was conducted in one Midwestern U.S. state; in some communities there are long standing ties as families may have lived there for many generations and there may have been concerns about personal and social repercussions. Messages that communicate to consumers the value of reporting may be effective if there is an understanding of how knowledge about illnesses can aid in preventing intentional and unintentional contamination of foods in the future. In this study, 36% of consumers were concerned or very concerned about food purchased to prepare at home and 57% were concerned or very concerned about food prepared away from home; these findings are similar to those in the 2011 NPR study of consumers which found 59% were concerned about the safety of food. About 68% of the healthcare providers participating in this study reported being concerned or very concerned about the safety of food; yet about 27% perceived that their clients or patients were. Of the 35 consumers in the focus groups in this study, 34% said they had become ill from something they ate in the last 3 months compared to 11% of the 3,017 consumers in the 2011 NPR study [10]. Of those who reported becoming ill, only one sought medical treatment compared to one third of those participants in the NPR study. Yet when asked if they had ever become ill from a food they had eaten, 32 reported yes and 11 said they had received medical treatment. Medical actions ranged from stool samples for 16 participants to assist with diagnosis and other treatments of the symptoms such as fluid intake. This U.S. state specific study also had similar findings to the national NPR study with identification of meat as a food category of concern by 77% of the consumer focus group respondents, second to seafood at 82% compared to meat by 44% of those in NPR study. Surprisingly, given national outbreaks and recalls of different fresh produce items, this food category was noted as a concern by about 30% of participants in both studies.

Lack of knowledge within the healthcare community about foodborne illness supports previous research conducted with healthcare professionals [16,17,18,20]. As the U.S. population increases in numbers that are considered at risk (such as the elderly), long lasting negative health impacts from a foodborne illness will have personal and social repercussions as well. Curriculum for RD eligibility includes food safety education focused on operational controls to mitigate risk of food borne illness. Yet is it not clear the extent of curricula requirements for other healthcare providers such as RN and medical doctor (MD). Findings from this study suggest healthcare providers who had some experiences with a food borne illness diagnosis were more aware of possibility symptoms presented may be food related. To offset these consequences, it is critical consumers and healthcare providers become more informed about the types of foodborne illness, the symptoms, the treatment protocols, and how should illnesses be reported. Because, patients trust their healthcare providers for food safety information [15] it is critical to increase healthcare professionals’ knowledge about foodborne illness and reporting. Participants in both groups identified common message content and delivery mechanisms that would achieve these goals. Content related to foodborne illness types, symptoms, suspected foods, stool sample protocol, and reporting process were identified. Some identified information needs were already available; findings suggest need to more effectively market availability of these resources. For consumers, an easy to identify report procedure and contact was identified with the suggestion of some type of toll free number, similar to a Poison Control Hotline that could be branded. Minnesota, another U.S. Midwestern state, found that a toll free complaint hotline was successful and aided in the detection of outbreaks [27]. The Minnesota hotline number is 1-877-FOOD-ILL [28].

Delivery mechanisms identified included a weekly email update of trends or upticks of illness in specific geographic areas. There appeared varying degrees of comfort with computer delivered instruction based on age and current infusion of technology into the workplace. For example, in one focus group the skills to fix the printer were considered as technological whereas on the other end of the continuum, there was a request for a smart phone application to assist with diagnosis of foodborne illness. However, a computer with internet access was readily available in workplaces; thus resources available for download and subsequent printing and posting as a referral was considered an acceptable method. 

Healthcare providers in this study appeared more focused on treatment of symptoms of the illness rather than confirming and reporting. This could be a result of lack of food safety knowledge, lack of time, or physician’s lack of understanding of the seriousness of foodborne illness [16]. An interesting theme that emerged was related to participants’ personal experiences as a victim of FBI or involvement in an investigation and treatment of such an illness. Those who identified past experiences appeared more inclined to recognize need for stool samples to diagnose a possible food related illness.
